# Case, &c.

**Published:** 1839-08-17

**Authors:** Thos. Miller

**Affiliations:** Washington


*Case, &c., communicated by* Thos. Miller, M. D.

*August 18th*, 1829.—Margaret T. (colored) aet. 25; house-maid, of good habits called upon me, stating that she was accustomed to have her water drawn off frequently, it being necessary for her comfort. I gathered from her the following particulars of her case, the greater portion of which has been confirmed by the testimony of several of her most respectable medical attendants. Several years previous to her first visit to me she had a typhus fever of long duration. After her recovery she experienced a difficulty in discharging her urine, which was attributed to the effects of blisters. The difficulty increased despite of all attempts at removing it, and it became necessary to resort to the catheter. She acquired the habit of drawing off the water herself, using the instrument for some time daily. After awhile the operation became less frequent, until a week or ten days would elapse without its performance. This state of affairs continued for eighteen months, when the patient found the passage of the catheter attended with so much pain, that she was forced to seek once more the aid of a physician. The instrument was then passed once in two weeks, afterwards once in three. Two years after the commencement of her troubles, she came to this city, and was successively advised by several of our practitioners. At the date above, she consulted me. She was then in the habit of passing the catheter once in six weeks.—Her general health appeared to have suffered but little. She had had no healthy catamenial discharge for several years—troubled with colicky pains which had become more frequent within the last year, attended by nausea and vomiting occuring regularly every morning. The discharge was watery, of an unpleasant urinous odour. It was sometimes tinged with blood. Her bowels were exceedingly torpid. There was constantly a urinous odour emanating from her person, which would be perceived in passing her upon the street. She perspired much more freely than ordinary individuals; her pulse 90, rather full; appetite capricious. Had a tumour excised from her sternum, which, from her description, and from the cicatrix, I judged to be schirrous. She offered the following symptoms when I examined her for the first time. Her abdomen hard, not much larger than natural, with a tympanitic sound over the region of the bladder; there was pain on pressure; with difficulty, and with suffering to the patient I passed my finger up the vagina. Immediately over the meatus urinarius I discovered a tumour as large as a hazle nut, hard and elastic; opposite to it another about the size of a partridge-egg; so that by these tumours the vagina was nearly blocked up. They were evidently similar in character to those taken from the sternum. There was in the vagina a tenacious fœtid acrid liquid, tinged with blood, which was constantly being discharged. The great difficulty in reaching the uterus, prevented me from ascertaining its source. I passed a male flexible catheter into the bladder. As soon as the point of the instrument passed beyond the tumours the patient was convulsed and insensible; even after the instrument was withdrawn, convulsion succeeded convulsion for five or six hours, so that I became alarmed for the patient's life. During the whole time that the convulsions lasted, the bladder remained open. I gave her medicine and directions, and left her. On the following morning I found her weak from the convulsions. She told me that she had not the faintest idea of any thing that passed after the catheter entered her urethra. The small quantity of urine which I drew off the day before surprised me—not more than half a pint, healthy in appearance, of strong odour. I treated the case for some time by various measures suggested by myself, and by several other medical gentlemen, without producing any satisfactory result. From November 1st, 1831, to April 1st, 1832, I lost sight of the case. On the last date she came to have her urine drawn off, stating that she had been quite regular in her catamenial discharge till within the last two months, when it had ceased. I was induced to believe she was pregnant. I delivered her of twins on December 21st, 1832; a few weeks previous to this event I passed the catheter for her. This was the last time I ever performed the operation for her. Her bladder from that time gradually resumed its functions, the discharge from the vagina with the tumours disappeared, she became *regular*, and within the last year she was delivered of another child. In this case I was often assisted by the advice of my friends Dr. W——, of the U. S. N. and Johnson. Several other physicians have witnessed the passage of the catheter and the effects produced, each time the phenomena mentioned above occurring.—The patient was always apprised of the necessity for the use of the instrument, by a sense of fulness in the pelvic region, pain in the loins, and a bearing-down feeling.

*Washington*, April, 1836.

